# 4-{(*E*)-2-[4-(But-3-en-1-yl­oxy)phen­yl]diazen-1-yl}benzoic acid

**DOI:** 10.1107/S1600536812038718

**Published:** 2012-09-19

**Authors:** Md. Lutfor Rahman, Huey Chong Kwong, Mashitah Mohd. Yusoff, Gurumurthy Hegde, Mohamed Ibrahim Mohamed Tahir, Mohamad Zaki. Ab. Rahman

**Affiliations:** a University Malaysia Pahang, Faculty of Industrial Sciences and Technology, 26300 Gambang, Kuantan, Pahang, Malaysia; b Department of Chemistry, Faculty Science, Universiti Putra Malaysia, 43400 UPM Serdang, Selangor, Malaysia

## Abstract

The title compound, C_17_H_16_N_2_O_3_, has an *E* conformation about the azobenzene (–N=N–) linkage. The benzene rings are twisted slightly with respect to each other [6.79 (9)°], while the dihedral angle between the plane through the carb­oxy group and the attached benzene ring is 3.2 (2)°. In the crystal, mol­ecules are oriented with the carb­oxy groups head-to-head, forming O—H⋯O hydrogen-bonded inversion dimers. These dimers are connected by C—H⋯O hydrogen-bonds into layers lying parallel to the (013) plane.

## Related literature

For the physical properties of compounds containing an azobenzene (–N=N–) linkage, see: Chigrinov (2005 ▶); Hegde (2007 ▶). For related structures, see: Yu & Liu (2009 ▶); Lai *et al.* (2002 ▶); Centore & Tuzi (2003 ▶). For standard bond lengths, see Allen *et al.* (1987 ▶).
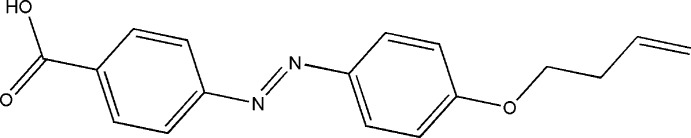


## Experimental

### 

#### Crystal data

C_17_H_16_N_2_O_3_
*M* *_r_* = 296.33
Triclinic, 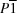

*a* = 7.0937 (7) Å
*b* = 9.8687 (10) Å
*c* = 11.2490 (11) Å
α = 87.334 (8)°
β = 73.475 (8)°
γ = 75.174 (8)°
*V* = 729.54 (13) Å^3^
*Z* = 2
Cu *K*α radiation
μ = 0.77 mm^−1^
*T* = 100 K
0.26 × 0.16 × 0.04 mm

#### Data collection

Oxford Diffraction Gemini diffractometer
Absorption correction: multi-scan (*CrysAlis RED*; Oxford Diffraction, 2006 ▶) *T*_min_ = 0.85, *T*_max_ = 0.97
9940 measured reflections
2783 independent reflections
2298 reflections with *I* > 2.0σ(*I*)
*R*_int_ = 0.024

#### Refinement

*R*[*F* ^2^ > 2σ(*F* ^2^)] = 0.054
*wR*(*F* ^2^) = 0.146
*S* = 1.00
2773 reflections
199 parameters
H-atom parameters constrained
Δρ_max_ = 0.51 e Å^−3^
Δρ_min_ = −0.33 e Å^−3^

### 

Data collection: *CrysAlis CCD* (Oxford Diffraction, 2006 ▶); cell refinement: *CrysAlis CCD*; data reduction: *CrysAlis RED* (Oxford Diffraction, 2006 ▶); program(s) used to solve structure: *Superflip* (Palatinus & Chapuis, 2007 ▶); program(s) used to refine structure: *CRYSTALS* (Betteridge *et al.*, 2003 ▶); molecular graphics: *Mercury* (Macrae *et al.*, 2006 ▶); software used to prepare material for publication: *CRYSTALS*.

## Supplementary Material

Crystal structure: contains datablock(s) global, I. DOI: 10.1107/S1600536812038718/su2476sup1.cif

Structure factors: contains datablock(s) I. DOI: 10.1107/S1600536812038718/su2476Isup2.hkl

Supplementary material file. DOI: 10.1107/S1600536812038718/su2476Isup3.cml

Additional supplementary materials:  crystallographic information; 3D view; checkCIF report

## Figures and Tables

**Table 1:** Hydrogen-bond geometry (Å, °)

*D*—H⋯*A*	*D*—H	H⋯*A*	*D*⋯*A*	*D*—H⋯*A*
O3—H2⋯O1^i^	0.87	1.76	2.612 (3)	166 (1)
C21—H211⋯O1^ii^	0.95	2.50	3.275 (3)	139 (1)

Symmetry codes: (i) 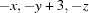; (ii) 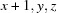.

**Fig. 1.**
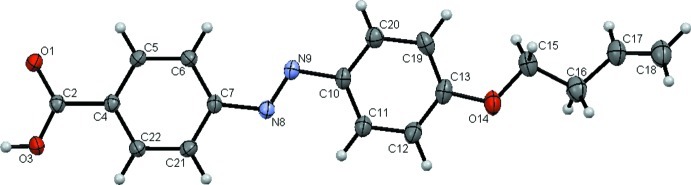
The molecular structure of the title molecule with the atom numbering and displacement ellipsoids drawn at the 50% probability level.

**Fig. 2.**
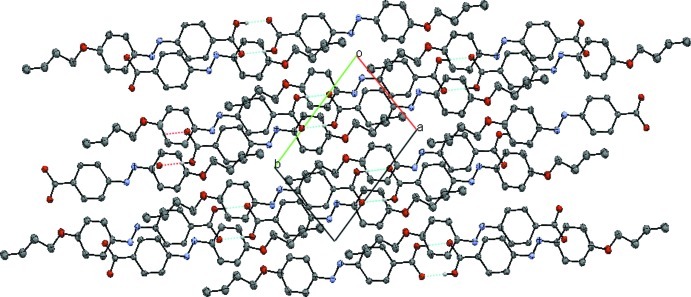
A view along the c axis of the crystal packing of the title compound, with the hydrogen bonds shown as dashed lines [the C-bound H atoms have been omitted for clarity].

## supplementary crystallographic information

### Comment

An in a molecule introduces the possibility of photochromism and photoisomerization (Chigrinov, 2005). Photonics, in which the light can be controlled by light as stimulus has been exploited (Hegde, 2007). Upon absorption of UV light (~ 365 nm) the energetically more stable E conformation, transforms into the *Z* conformation. The reverse transformation of the *Z* isomer into the E isomer can be brought about by irradiation with visible light (in the range of 400–500 nm). The latter can also occur in the "dark" by a process known as "thermal back relaxation" in a period ranging from minutes to tens of hours depending on the system. In this case molecules again transform from the metastable *cis*-conformation to the energetically stable *trans*-conformation. In conclusion, the present investigation on rod-shaped azo dyes is very useful for a variety of photonic applications. Excellent quality, cost effective, easy to prepare, are properties which make these devices very attractive for future generations. Detailed investigations on the physics of these azo dyes is under intense consideration.

The bond lengths (Allen *et al.*,1987) and bond angles in the titled compound (Fig. 1) are normal. The carbonyl group (C2/O1/O3) is almost coplanar with the attached benzene ring (C4-C7/C21/C22) with a dihedral angle of only 3.2 (2)°. The length of N8═N9 bond is 1.263 (2) Å and the torsion angle for the azo unit (C7—N8—N9—C10) is -177.75 (16)° rather than ca. ±180° as observed elsewhere: For example: 4,4-Azinodibenzoic acid (Yu and Liu, 2009) and (*E*)-ethyl 4-((4-(decanoyloxy)phenyl)diazenyl)benzoate (Lai *et al.*,2002). However, it is comparable with the value of 175.10° observed for (*E*)-4-((4-((2-hydroxyethyl)(methyl)amino)phenyl)diazenyl)benzoic acid (Centore & Tuzi, 2003). The benzene rings (C4-C7/C21/C22 and C10-C3/C19/C20) lie at a mutual dihedral angles of 6.79 (9)°, compared to 16.69° in (*E*)-4-((4-((2-hydroxyethyl)(methyl)amino)phenyl)diazenyl)benzoic acid (Centore & Tuzi, 2003). The C15—C16—C17—C18 torsion angle in the butyl group is 126.1 (3)°.

In the crystal, the carboxyl groups are oriented head-to-head forming hydrogen bonded inversion dimers (Table 1 and Fig. 2). These dimers are further linked by C—H···O hydrogen bonds to a generate a layer parallel to the (013) plane (Table 1 and Fig. 2).

### Experimental

The title compound was prepared from ethyl 4-aminobenzoate. Firstly the diazonuim salt was prepared using one equivalent of sodium nitrite to one equivalent of ethyl 4-aminobenzoate in methanol - water mixture at 275 K, in the presence of 3 equivalents of aqueous hydrochloric acid, which was coupled with phenol to yield ethyl 4-[(4-hydroxyphenyl)diazenyl]benzoate. This compound was then alkylated with 4-bromo-1-butene in the presence of potassium carbonate as base to give the ester, ethyl 4-{[4-(but-3-en-1-yloxy)phenyl]diazenyl}benzoate. This compound was then hydrolyzed under basic conditions to yield the title benzoic acid. Brown plate-like crystals of the title compound were obtained by slow evaporation of a solution in methanol.

### Refinement

The H atoms were all located in a difference Fourier map, but those attached to carbon atoms were repositioned geometrically. They were all initially refined with soft restraints on the bond lengths and angles to regularize their geometry: C—H = 0.93 (2)–0.98 (2) Å and O—H = 0.82 (2) Å with *U*_iso_(H) = k × *U*_eq_(O,C) where k = 1.5 for the OH H atom and = 1.2 for the C-bound H atoms. In the final cycles or refinement they were allowed to ride on their parent atom.

### Crystal data

**Table:**

C_17_H_16_N_2_O_3_	*Z* = 2
*M**_r_* = 296.33	*F*(000) = 312
Triclinic, *P*1	*D*_x_ = 1.349 Mg m^−^^3^
Hall symbol: -P 1	Cu *K*α radiation, λ = 1.54180 Å
*a* = 7.0937 (7) Å	Cell parameters from 3768 reflections
*b* = 9.8687 (10) Å	θ = 4–71°
*c* = 11.2490 (11) Å	µ = 0.77 mm^−^^1^
α = 87.334 (8)°	*T* = 100 K
β = 73.475 (8)°	Plate, brown
γ = 75.174 (8)°	0.26 × 0.16 × 0.04 mm
*V* = 729.54 (13) Å^3^	

### Data collection

**Table:**

Oxford Diffraction Gemini diffractometer	2783 independent reflections
Radiation source: sealed x-ray tube	2298 reflections with *I* > 2.0σ(*I*)
Graphite monochromator	*R*_int_ = 0.024
ω scans	θ_max_ = 71.5°, θ_min_ = 4.1°
Absorption correction: multi-scan (*CrysAlis RED*; Oxford Diffraction, 2006)	*h* = −8→8
*T*_min_ = 0.85, *T*_max_ = 0.97	*k* = −11→11
9940 measured reflections	*l* = −13→13

### Refinement

**Table:**

Refinement on *F*^2^	Primary atom site location: structure-invariant direct methods
Least-squares matrix: full	Hydrogen site location: difference Fourier map
*R*[*F*^2^ > 2σ(*F*^2^)] = 0.054	H-atom parameters constrained
*wR*(*F*^2^) = 0.146	Method = Modified Sheldrick *w* = 1/[σ^2^(*F*^2^) + ( 0.06*P*)^2^ + 0.76*P*], where *P* = (max(*F*_o_^2^,0) + 2*F*_c_^2^)/3
*S* = 1.00	(Δ/σ)_max_ = 0.0003776
2773 reflections	Δρ_max_ = 0.51 e Å^−^^3^
199 parameters	Δρ_min_ = −0.33 e Å^−^^3^
0 restraints	

### Special details

**Table:**

Refinement. This compound, 9940 numbers of reflections were collected and measured during the refinement. Symmetry related reflections were measured more than once and after merging the symmetry equivalent reflections there were only 2783 reflection left. 10 more reflections were filtered, as sigma cutoff was set as 3 and (sin*θ*/x)set to>0.01 (to eliminate reflection measured near the vicinity of beam stop) therefore numbers of reflection reduced to 2773.

### Fractional atomic coordinates and isotropic or equivalent isotropic displacement parameters (Å^2^)

**Table:**

	*x*	*y*	*z*	*U*_iso_*/*U*_eq_	
O1	0.0122 (2)	1.34005 (15)	0.07329 (14)	0.0276	
C2	0.1938 (3)	1.3516 (2)	0.04699 (18)	0.0221	
O3	0.2517 (2)	1.45464 (14)	−0.00910 (14)	0.0281	
C4	0.3484 (3)	1.2387 (2)	0.08338 (17)	0.0215	
C5	0.2990 (3)	1.1180 (2)	0.14000 (18)	0.0231	
C6	0.4449 (3)	1.0125 (2)	0.17190 (18)	0.0233	
C7	0.6447 (3)	1.0260 (2)	0.14525 (18)	0.0220	
N8	0.8099 (2)	0.92608 (18)	0.17206 (15)	0.0238	
N9	0.7751 (2)	0.81065 (17)	0.21222 (15)	0.0235	
C10	0.9450 (3)	0.7184 (2)	0.24132 (18)	0.0243	
C11	1.1313 (3)	0.7536 (2)	0.2247 (2)	0.0293	
C12	1.2896 (3)	0.6624 (2)	0.2565 (2)	0.0321	
C13	1.2665 (3)	0.5349 (2)	0.30679 (19)	0.0302	
O14	1.4341 (2)	0.45519 (16)	0.33669 (15)	0.0364	
C15	1.4290 (4)	0.3232 (2)	0.3930 (2)	0.0349	
C16	1.6307 (4)	0.2701 (3)	0.4247 (2)	0.0399	
C17	1.6430 (4)	0.1366 (3)	0.4911 (2)	0.0388	
C18	1.7890 (4)	0.0213 (3)	0.4581 (3)	0.0455	
C19	1.0850 (3)	0.4962 (2)	0.32338 (19)	0.0310	
C20	0.9240 (3)	0.5897 (2)	0.28909 (19)	0.0287	
C21	0.6931 (3)	1.1467 (2)	0.09065 (19)	0.0252	
C22	0.5462 (3)	1.2529 (2)	0.06039 (18)	0.0238	
H51	0.1631	1.1098	0.1566	0.0298*	
H61	0.4112	0.9298	0.2120	0.0302*	
H111	1.1483	0.8433	0.1888	0.0379*	
H121	1.4168	0.6860	0.2448	0.0406*	
H151	1.3110	0.3369	0.4683	0.0453*	
H152	1.4146	0.2584	0.3328	0.0447*	
H161	1.6418	0.3468	0.4751	0.0512*	
H162	1.7478	0.2531	0.3482	0.0518*	
H171	1.5325	0.1356	0.5657	0.0520*	
H182	1.9017	0.0237	0.3829	0.0605*	
H181	1.7859	−0.0632	0.5068	0.0604*	
H191	1.0658	0.4083	0.3577	0.0394*	
H201	0.8003	0.5652	0.2975	0.0369*	
H211	0.8278	1.1552	0.0740	0.0331*	
H221	0.5794	1.3355	0.0213	0.0322*	
H2	0.1704	1.5151	−0.0423	0.0500*	

### Atomic displacement parameters (Å^2^)

**Table:**

	*U*^11^	*U*^22^	*U*^33^	*U*^12^	*U*^13^	*U*^23^
O1	0.0212 (7)	0.0239 (8)	0.0399 (8)	−0.0047 (6)	−0.0132 (6)	0.0029 (6)
C2	0.0228 (10)	0.0191 (10)	0.0252 (10)	−0.0034 (8)	−0.0092 (8)	−0.0018 (8)
O3	0.0270 (8)	0.0210 (8)	0.0385 (8)	−0.0048 (6)	−0.0154 (6)	0.0082 (6)
C4	0.0213 (10)	0.0195 (10)	0.0243 (9)	−0.0033 (8)	−0.0087 (8)	−0.0015 (8)
C5	0.0183 (9)	0.0230 (11)	0.0295 (10)	−0.0048 (8)	−0.0094 (8)	0.0016 (8)
C6	0.0236 (10)	0.0203 (10)	0.0279 (10)	−0.0064 (8)	−0.0098 (8)	0.0030 (8)
C7	0.0213 (10)	0.0198 (10)	0.0248 (10)	−0.0009 (8)	−0.0100 (8)	−0.0005 (8)
N8	0.0217 (8)	0.0225 (9)	0.0272 (9)	−0.0028 (7)	−0.0094 (7)	0.0016 (7)
N9	0.0207 (8)	0.0218 (9)	0.0258 (8)	−0.0003 (7)	−0.0073 (7)	−0.0008 (7)
C10	0.0235 (10)	0.0231 (11)	0.0228 (10)	0.0018 (8)	−0.0079 (8)	−0.0014 (8)
C11	0.0256 (11)	0.0273 (11)	0.0338 (11)	−0.0009 (9)	−0.0116 (9)	−0.0001 (9)
C12	0.0260 (11)	0.0317 (12)	0.0391 (12)	−0.0018 (9)	−0.0145 (9)	−0.0008 (10)
C13	0.0281 (11)	0.0311 (12)	0.0276 (10)	0.0047 (9)	−0.0119 (9)	−0.0046 (9)
O14	0.0356 (9)	0.0287 (8)	0.0461 (9)	0.0007 (7)	−0.0216 (7)	0.0028 (7)
C15	0.0410 (13)	0.0270 (12)	0.0348 (12)	−0.0002 (10)	−0.0149 (10)	−0.0006 (9)
C16	0.0456 (14)	0.0359 (14)	0.0420 (13)	−0.0055 (11)	−0.0228 (11)	0.0019 (10)
C17	0.0383 (13)	0.0374 (13)	0.0408 (13)	−0.0019 (10)	−0.0187 (10)	0.0032 (10)
C18	0.0463 (15)	0.0372 (14)	0.0563 (16)	−0.0033 (11)	−0.0264 (13)	0.0022 (12)
C19	0.0390 (12)	0.0218 (11)	0.0273 (10)	0.0000 (9)	−0.0086 (9)	0.0013 (8)
C20	0.0272 (11)	0.0270 (11)	0.0284 (11)	−0.0022 (9)	−0.0064 (8)	−0.0009 (8)
C21	0.0196 (9)	0.0265 (11)	0.0310 (10)	−0.0066 (8)	−0.0087 (8)	0.0002 (8)
C22	0.0237 (10)	0.0196 (10)	0.0294 (10)	−0.0055 (8)	−0.0101 (8)	0.0031 (8)

### Geometric parameters (Å, º)

**Table:**

O1—C2	1.271 (2)	C13—O14	1.367 (3)
C2—O3	1.268 (2)	C13—C19	1.395 (3)
C2—C4	1.483 (3)	O14—C15	1.426 (3)
O3—H2	0.868	C15—C16	1.529 (3)
C4—C5	1.401 (3)	C15—H151	0.993
C4—C22	1.395 (3)	C15—H152	0.996
C5—C6	1.382 (3)	C16—C17	1.479 (3)
C5—H51	0.952	C16—H161	0.998
C6—C7	1.403 (3)	C16—H162	0.997
C6—H61	0.965	C17—C18	1.312 (4)
C7—N8	1.421 (3)	C17—H171	0.974
C7—C21	1.391 (3)	C18—H182	0.989
N8—N9	1.263 (2)	C18—H181	0.978
N9—C10	1.423 (3)	C19—C20	1.408 (3)
C10—C11	1.411 (3)	C19—H191	0.959
C10—C20	1.382 (3)	C20—H201	0.947
C11—C12	1.371 (3)	C21—C22	1.382 (3)
C11—H111	0.977	C21—H211	0.945
C12—C13	1.384 (3)	C22—H221	0.959
C12—H121	0.960		
			
O1—C2—O3	123.76 (18)	O14—C15—C16	106.04 (19)
O1—C2—C4	118.68 (17)	O14—C15—H151	109.0
O3—C2—C4	117.56 (17)	C16—C15—H151	111.8
C2—O3—H2	119.6	O14—C15—H152	108.8
C2—C4—C5	121.02 (17)	C16—C15—H152	111.3
C2—C4—C22	119.37 (17)	H151—C15—H152	109.7
C5—C4—C22	119.61 (18)	C15—C16—C17	112.4 (2)
C4—C5—C6	120.55 (18)	C15—C16—H161	106.1
C4—C5—H51	119.1	C17—C16—H161	112.0
C6—C5—H51	120.4	C15—C16—H162	111.1
C5—C6—C7	119.42 (18)	C17—C16—H162	107.0
C5—C6—H61	120.9	H161—C16—H162	108.3
C7—C6—H61	119.7	C16—C17—C18	125.6 (3)
C6—C7—N8	125.56 (18)	C16—C17—H171	116.3
C6—C7—C21	119.98 (18)	C18—C17—H171	118.0
N8—C7—C21	114.44 (17)	C17—C18—H182	117.5
C7—N8—N9	115.89 (16)	C17—C18—H181	120.8
N8—N9—C10	112.56 (16)	H182—C18—H181	121.7
N9—C10—C11	122.76 (18)	C13—C19—C20	118.9 (2)
N9—C10—C20	118.03 (18)	C13—C19—H191	122.3
C11—C10—C20	119.21 (19)	C20—C19—H191	118.8
C10—C11—C12	120.7 (2)	C19—C20—C10	120.4 (2)
C10—C11—H111	119.5	C19—C20—H201	120.6
C12—C11—H111	119.8	C10—C20—H201	118.9
C11—C12—C13	119.9 (2)	C7—C21—C22	120.46 (18)
C11—C12—H121	120.8	C7—C21—H211	119.3
C13—C12—H121	119.3	C22—C21—H211	120.3
C12—C13—O14	114.02 (19)	C4—C22—C21	119.94 (18)
C12—C13—C19	120.84 (19)	C4—C22—H221	119.3
O14—C13—C19	125.1 (2)	C21—C22—H221	120.7
C13—O14—C15	119.93 (18)		

### Hydrogen-bond geometry (Å, º)

**Table:**

*D*—H···*A*	*D*—H	H···*A*	*D*···*A*	*D*—H···*A*
O3—H2···O1^i^	0.87	1.76	2.612 (3)	166 (1)
C21—H211···O1^ii^	0.95	2.50	3.275 (3)	139 (1)

Symmetry codes: (i) −*x*, −*y*+3, −*z*; (ii) *x*+1, *y*, *z*.
